# Large-scale *ex vivo* generation of human neutrophils from cord blood CD34^+^ cells

**DOI:** 10.1371/journal.pone.0180832

**Published:** 2017-07-11

**Authors:** Zhenwang Jie, Yu Zhang, Chen Wang, Bin Shen, Xin Guan, Zhihua Ren, Xinxin Ding, Wei Dai, Yongping Jiang

**Affiliations:** 1 Biopharmaceutical R&D Center, Peking Union Medical College of Tsinghua University, Suzhou, China; 2 Biopharmagen corp., Suzhou, China; 3 College of Nanoscale Science and Engineering, SUNY Polytechnic Institute, Albany, New York City, United States of America; 4 Environmental Medicine, NYU Langone Medical Center, Tuxedo, New York City, United States of America; Universiti Putra Malaysia, MALAYSIA

## Abstract

Conventional high-dose chemotherapy frequently leads to severe neutropenia, during which patients experience a high risk of infection. Although support care with donor’s neutrophils is possible this choice is largely hampered by the limited availability of matched donors. To overcome this problem, we explored a large-scale *ex vivo* production of neutrophils from hematopoietic stem cells (HSCs) using a four-stage culture approach in a roller-bottle production platform. We expanded CD34^+^ HSCs isolated from umbilical cord blood (UCB) using our in-house special medium supplemented with cytokine cocktails and achieved about 49000-fold expansion of cells, among which about 61% were differentiated mature neutrophils. *Ex vivo* differentiated neutrophils exhibited a chemotactic activity similar to those from healthy donors and were capable of killing *E*. *coli in vitro*. The expansion yield as reported herein was at least 5 times higher than any other methods reported in the literature. Moreover, the cost of our modified medium was only a small fraction (<1/60) of the StemSpan^™^ SFEM. Therefore, our *ex vivo* expansion platform, coupled with a low cost of stem cell culture due to the use of a modified medium, makes large-scale manufacturing neutrophils possible, which should be able to greatly ameliorate neutrophil shortage for transfusion in the clinic.

## Introduction

Neutrophils are special phagocytes that are found in the bloodstream. During the beginning or acute phase of inflammation, particularly as a result of bacterial infection, environmental exposure [1], and tumorigenesis [2, 3], neutrophils are among the first-responders of inflammatory cells that migrate towards the site of inflammation. In the clinic, patients who undergo extensive chemotherapy often experience frequent and prolonged periods of neutropenia, a major risk factor for severe bacterial and fungal infection [4] [5]. Despite the use of modern antibiotics and/or hematopoietic growth factors to shorten the period of treatment-induced neutropenia, infection remains the major cause of morbidity and mortality in these patients [6]. For typical leukemic patients who receive chemotherapy and subsequent bone marrow transplantation, there is a gap about 8 to 12 days of severe neutropenia before their neutrophil counts return to the normal (0.5 x 10^9^ neutrophils/L) [7]. G-CSF is often ineffective for some patients with a loss of bone marrow function. Moreover, there are fungal or bacterial infections that are unresponsive to antimicrobial treatments as demonstrated by visible spreading lesions on skin, mucosa or radiological examination [8].

To date, neutrophil transfusion is the only logical approach to the treatment of infections in neutropenic patients. Haylock and colleagues [9] have proposed that administration of *ex vivo*-expanded neutrophil precursors can shorten the neutropenic period, thus reducing the risk of infection. Methods for collecting cells from normal donors have been available for more than 25 years. However, due to a lack of an effective method for obtaining HSC-derived neutrophils with high purity and quantity, successful clinical transfusion of *ex vivo* expanded neutrophils has not been achieved. In the past, a few research groups have obtained immortalized neutrophil cell lines from induced pluripotent stem (iPS) cells [10–12]. However, due to safety concern, iPS cell-derived neutrophils have not been used for clinical applications. It has been proposed that *ex vivo* expanded neutrophils from CD34^+^ hematopoietic stem cells can be used as an autologous source of cells for transplantation because of their ease of collection and less stringent HLA matching, as well as a high rate of cell proliferation [9]. In fact, several groups have obtained *ex vivo*-expanded CD34^+^ which are granulocyte-specific [13–16]. Moreover, small clinical studies were performed using human cord blood-derived CD34^+^ cells in patients receiving chemotherapy, which markedly reduced the recovery period of granulocyte counts [17–19].

The major obstacle of generating neutrophils *ex vivo* is that CD34^+^ cells are not efficiently expanded before inducing them to mature neutrophils. Several research groups have tried to produce neutrophils from CD34^+^ cells (from a single UCB collection which yields about 5x10^6^ CD34^+^ cells) that were not sufficiently expanded [20, 21]. Currently known methods for a large scale *ex vivo* expansion are at best capable of generating two doses of clinical neutrophils, which are roughly equivalent to 10,000-fold expansion. Although increasing the amount of starting cells from mobilized peripheral blood can potentially generate up to ten doses of neutrophils [22] the total neutrophils generated through this approach are only sufficient for a single treatment per donation.

In this report, we describe an optimized four-stage culture approach using our in-house culture medium and the roller-bottle production platform that can generate neutrophils *ex vivo* on a large scale. We believe that our new stem cell expansion and differentiation platform is capable of providing large amounts of high quality neutrophils for clinical applications.

## Materials and methods

### Ethics statement

All studies that involved the use of animals were conducted according to relevant national and international guidelines. Both male and female NOD/SCID mice of 6–8 weeks of age were purchased from the Shanghai Laboratory Animal Co (SLAC, Shanghai, China, http://www.slaccas.com/). Experiment protocols were approved by the Institutional Animal Care and Use Committees of Soochow University [IACUC permit number: SYXK(Su) 2013–0018], and were in accordance with the Guidelines for the Care and Use of Laboratory Animals (National Research Council, People’s Republic of China, 2012). We further attest that all efforts were made to ensure minimal animal suffering. All fresh UCB samples were provided with a written consent from volunteer patients at Suzhou Municipal Hospital (Suzhou, China). Consent forms were signed by participated patients. The overall study and all necessary signed forms were approved by the Hospital's Ethics Committee and Research Ethics Advisory Committee.

### Cytokines, antibodies, and reagents

Recombinant human stem cell factor (SCF), fms-related tyrosine kinase 3 ligand (Flt-3L), granulocyte colony-stimulating factor(G-CSF), granulocyte-macrophage colony-stimulating factor (GM-CSF), interleukin (IL)-3, thrombopoietin (TPO) and insulin were purchased from Biopharmagen Corp (Suzhou, China, http://www.biopharmagen.com/). IL-1β and IL-8 were purchased from PeproTech Inc. (Rocky Hill, NJ; http://www.peprotech.com). Fluorescein isothiocyanate (FITC)-conjugated anti-CD66b antibody, FITC-conjugated anti-38 antibody, Allophycocyanin (APC) and phycoerythrin (PE) -conjugated anti-CD34 antibody, and FITC-conjugated anti-CD45 antibody were purchased from BD Biosciences (San Diego, CA, http://www.bdbiosciences.com). Putrescine, selenium, transferrin, zymosan, HEPES, human AB serum, Hanks’ balanced saline solution (HBSS) and formyl-methionyl-leucyl-phenylalanine (FMLP) were from Sigma-Aldrich (St. Louis, MO, http://www.sigmaaldrich.com). B-27 supplements were from Thermo Fisher Scientific Life Sciences (Waltham, MA, http://www.thermofisher.com).

### Collection of CD34^+^ cells

Fresh UCB samples were obtained within 6–8 hours of delivery from Suzhou Municipal Hospital (Suzhou, China) with written consents from donors. The protocol was approved by the Suzhou Municipal Hospital Ethics Committee and Research Ethics Advisory Committee. Mononuclear cells were isolated by Ficoll-Paque (GE Healthcare, Marlborough, MA, http://www.gehealthcare.com) density gradient centrifugation [23]. CD34^+^ cells were enriched from mononuclear cells by magnetic cell sorting using a MACS Direct CD34 MicroBead Kit (Miltenyi Biotec, Amsterdam, The Netherlands, http://www.miltenyibiotec.com) per manufacturer’s instructions. Enriched CD34^+^ cells with purity ranging from 91% to 98% were confirmed by flow cytometry (BD Biosciences) after staining with a PE-conjugated anti-CD34 antibody.

### Cell culture

Isolated CD34^+^ cells were cultured and expanded in 25-T flasks (Corning, USA). After 6 days’ culture, expanded cells were cultured in a supplement bottle turning device (HERAcell 240i, Thermo Fisher Scientific, USA). Culture bottles were placed horizontally in an incubator at 37°C with 5% CO_2_ in the air with a rotation rate set at 0.82U per minute(min). Cells were cultured in a modified medium based on Iscove’s modified Dulbecco’s medium (IMDM) (Life Technologies, USA) after the addition of nutrition supplements [24, 25] consisting of putrescine (100 μM), selenium (5 ng/mL), insulin (25 μg/mL), transferrin (50 μg/mL), and B-27 Supplements (2%, v/v). A staged culture protocol was designed for *ex vivo* expansion and differentiation, which included Stage 1 (days 0–6), Stage 2 (days 7 ~9), Stage 3 (days 10~15), and Stage 4 (days16~18), respectively, fresh cytokines are replaced every three days. To optimize progenitor cell proliferation and neutrophil differentiation, different combinations of growth factors and cytokines, including SCF, Flt-3L, G-CSF, GM-CSF, IL-3, TPO, and fetal bovine serum (FBS) (10%, v/v, Hyclone, USA), were supplemented to modified culture media at various concentrations (Table 1). Culture optimization was carried out in a 2L-bottle with 200 ml medium. Once the optimal culture condition was achieved and finalized, Take three independent cord blood samples as described above for CD34^+^ hematopoietic stem cell separation method, part of isolated CD34^+^ cells(5×10^5^) were cultured and expanded in 25-T flasks. After 6 days’ culture, expanded cells were cultured in the same-size bottle containing 600 ml medium. Cells were sub-cultured and cryopreserved to maintain an optimal cell density which ranged from 2×10^5^ to 1×10^6^ cells/ml [26].

**Table 1:** Culture formula optimization for human neutrophil cells expansion.

Group		IMDM	Nutrition Supplements	FBS (10%, V/V)	HSA (1%,V/V	SCF (ng/mL)	G-CSF (ng/mL)	IL-3 (ng/mL)	FL (ng/mL)	GM-CSF (ng/mL)	TPO (ng/mL)
Stage 1	IMDM	+	-	-	-	-	-	-	-	-	-
	MN	+	+	-	-	-	-	-	-	-	-
	MNF	+	+	+	-	-	-	-	-	-	-
	MNF+SFGM3T(T5)	+	+	+	-	100	50	25	100	15	5
	MNF+SFGM3T(T50)	+	+	+	-	100	50	25	100	15	50
	MNF+SFGM3T(GM5)	+	+	+	-	100	50	25	100	5	20
	MNF+SFGM3T(GM50)	+	+	+	-	100	50	25	100	50	20
	MNF+SFGM3T^※^	+	+	+	-	100	50	25	100	15	20
	MN+SFGM3T	+	+	-	-	100	50	25	100	15	20
Stage 2	MN	+	+	-	-	-	-	-	-	-	-
	MNF	+	+	+	-	-	-	-	-	-	-
	MNF+SFGM3(GC50)	+	+	+	-	100	50	15	100	10	-
	MNF+SFGM3(GC100)	+	+	+	-	100	100	15	100	10	-
	MNF+SFGM3(GM5)	+	+	+	-	100	75	15	100	5	-
	MNF+SFGM3(GM20)	+	+	+	-	100	75	15	100	20	-
	MNF+SFGM3^※^	+	+	+	-	100	75	15	100	10	-
	MN+SFGM3	+	+	-	-	100	75	15	100	10	-
Stage 3	MN	+	+	-	-	-	-	-	-	-	-
	MNF	+	+	+	-	-	-	-	-	-	-
	MNF+SFG(GC500)	+	+	+	-	100	500	-	100	-	-
	MNF+SFG(GC400)	+	+	+	-	100	400	-	100	-	-
	MNF+SFG(GC300)	+	+	+	-	100	300	-	100	-	-
	MNF+SFG(GC200)	+	+	+	-	100	200	-	100	-	-
	MNF+SFG^※^	+	+	+	-	100	100	-	100	-	-
	MN+SFG	+	+	-	-	100	100	-	100	-	-
Stage 4	MNA+SFG^※^	+	+	-	+	100	100	-	100	-	-

^※^ The final optimized media formula in each stage.

Cell numbers were determined at various time points using the Count Star^®^ cell counting system (Ailite, China) and were stained with Wright-Giemsa reagents (NJJCBIO, Nanjing, China) for morphological analysis. Images of stained cells were taken under a microscope with a digital camera (Olympus, Tokyo, Japan) at 400x magnification. Neutrophils were identified and quantified with a FACSVerse flow cytometer (BD Biosciences) after staining with an anti-CD66b antibody according to the procedure described in Saeki et al [27].

### In vitro bacterial killing assay

*Escherichia coli* BL21 was picked from single colonies grown on LB-agar plates, inoculated into LB broth and grown for 18h at 37°C. Microorganisms were pelleted by centrifugation at 2000g for 5 min, transferred into a microtube, washed once in 0.9% NaCl solution by centrifugation at 12,000g for 10s, and suspended in 1.5ml 0.9% NaCl solution. Bacterial concentration was determined by measurement of turbidity at 500nm. Suspensions were diluted to 1×10^8^ cells/ml in 1 ml medium [28], HEPES-buffered saline with 10% human AB serum and opsonized for 30 min at 37°C in a shaking water bath. Opsonized bacteria were kept on ice until use. Neutrophils were suspended in 100μl HEPES-buffered saline with 40% human AB serum at a concentration of 5×10^6^/ml. The opsonized *E coli* were added to the suspension of neutrophils at a neutrophil/bacterium ratio of 2:1. After incubation for 1 h, 50μL of aliquots with and without neutrophils were diluted in 2.5 ml alkalinized water (pH 11) for neutrophils lysis. All the samples were then inverted twice. After standing for 5 min at room temperature and then vortex vigorously for 5 s, 50 μl of the samples were diluted in PBS to achieve a bacterial concentration of about 2×10^3^/ml. Ten microliter aliquots of the diluted suspensions were spread on LB with 1.5% agar. Bacterial colonies were counted after overnight incubation.

### Chemotaxis assay

Chemotactic ability was determined using a modified Boyden chamber method as described [29] [30]. Chemotaxis was assessed using Transwell (3-μm pore size; Corning Inc., Corning, NY 14831 USA, http://www.corning.com/cn/zh/products/life-sciences.html), which was placed in a 24-well dish. The lower chamber, a 24-well culture dish, was filled with 500 μl Hanks’ balanced saline solution (HBSS) per well supplemented with 2.5% FBS whereas the upper chamber, a chemotaxel cup, was filled with *ex-vivo* differentiated neutrophils (EDN) or human peripheral blood-derived neutrophils (PBN) suspended in 500μl of HBSS per well supplemented with 2.5% FBS (4×10^5^ cells per milliliter). As chemoattractants, 100nM formyl-methionyl-leucyl-phenylalanine (FMLP) and 10ng/ml IL-8 were added to lower chambers. After incubation at 37°C for 4 h, cells from three randomly selected areas of the lower chamber were counted. In the meantime, cells in the chemotaxel cup were fixed and stained with the CD66b antibody for flow cytometer analysis.

### Transplantation of human UCB-derived neutrophils into mice and air-pouch chemotaxis assay

Eight-week old female NOD/SCID mice were made neutropenic by a single intraperitoneal injection of 5-fluorouracil (5-FU; 150mg/kg). Air pouches were produced by subcutaneous injection of 10 ml sterile air into the back of female mice [31]. Three days later, 5ml sterile air was injected into the same cavity. After another 3 days, mice received *i*.*v*. transplants of 2× 10^6^ *ex-vivo* differentiated neutrophils (EDN) or human peripheral blood-derived neutrophils (PBN) or vehicle (saline). Five hundred microliters of PBS with or without Zymosan (1mg/ml) and human IL-1β (10 ng/ml) were injected into the air pouch. Sixteen hour post injection, mice were sacrificed with 1% pentobarbital (50 mg/kg), and the air pouch was washed with 1 ml of ice-cold PBS to obtain cumulated leukocytes, which were subsequently confirmed by flow cytometric analysis after staining with the anti-CD66b^+^ cells [32].

### In vivo safety and function study in NOD/SCID mouse

The safety and function study of mice *in vivo* experiments using the method of Guan and colleges [33]. NOD/SCID mice were intravenously administered with 1x10^7^ human UCB-derived, differentiated neutrophils (day 18 culture as above), human peripheral blood neutrophils (PBN), or saline after sub-lethal irradiation (2.5Gy). Mouse peripheral blood samples were then collected from the retro-orbital plexus at different times post transplantation and stained with antibodies against human CD66b and CD45 followed by flow cytometric analysis. Human neutrophil progenitor cells cultured on the ninth day which contains 3.2×10^5^ CD34^+^ cells were also injected via tail vein into irradiated mice, after which mouse PB samples were collected and human neutrophil cells were determined by flow cytometry as above. Mouse bone marrow was harvested from both femurs 2 months after transplantation. Hematopoietic stem cells (CD34^+^), mature neutrophils (CD66b^+^) and promyelocytes (CD38^+^) [34] in bone marrow were determined by flow cytometry [35].

### Statistical analysis

One-way analysis of variance (ANOVA) followed by Dunnett’s multiple comparison tests was used for comparison among the various treatment groups. Results were considered statistically significant when the P value was less than 0.05.

## Results

### Culture optimization for ex vivo generation of neutrophils from UCB CD34^+^ cells

We first designed and optimized a four-stage culture protocol for *ex vivo* expansion and differentiation of neutrophils from human UCB CD34^+^ cells (Table 1). Distinct culture medium formulas as described were used for each stage.

**Fig 1:**
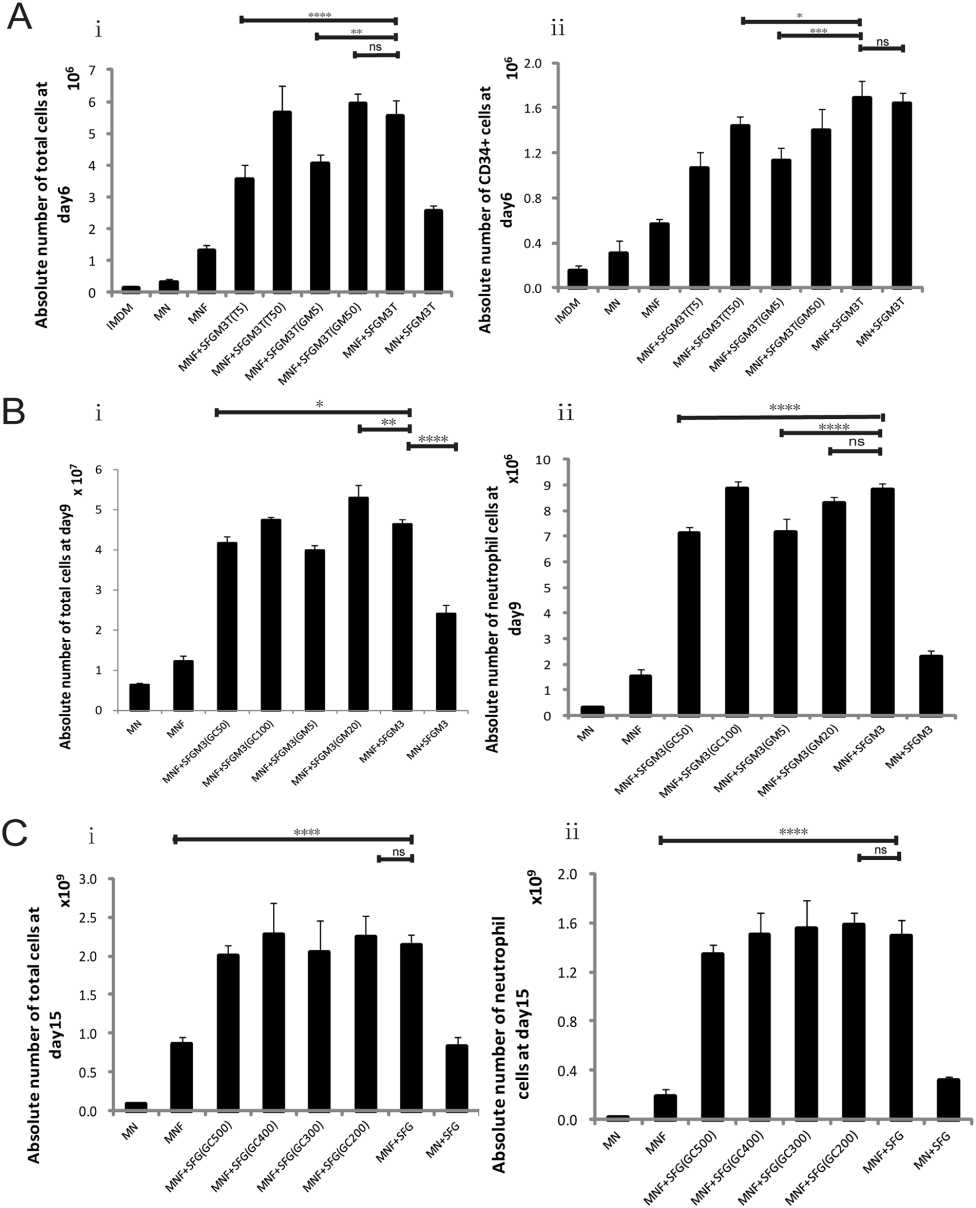
Optimization of culture conditions for *ex vivo* expansion of human neutrophils from human UCB CD34^+^ cells. (A) Absolute cell numbers were calculated on a particular day (e.g., 5×10^4^ CD34^+^ cells seeded on day 0). In Stage 1, isolated CD34^+^ cells were cultured for six days in various medium formulas (Table 1), absolute numbers of the total cell [A(i)] and CD34^+^ cell [A(ii)] were calculated on day 6. (B) Stage 2 started with the cells derived from group MNF+SFGM3T (IMDM +Nutrition Supplements **+** 100ng/ml SCF + 100 ng/ml Flt-3L + 50ng/ml G-CSF + 20ng/ml TPO + 25ng/ml IL-3 +15ng/mL GM-CSF) of Stage 1. Absolute numbers of total cells [B(i)], CD66b^+^ cell [B(ii)] were calculated on day 9 with G-CSF ranging from 50 to 100 ng/ml and GM-CSF ranging from 5 to 20 ng/ml in MNF+SFGM3 medium (IMDM + nutrition supplements + FBS + 100 ng/ml SCF + 100 ng/ml Flt-3L + 75 ng/ml G-CSF + 15 ng/ml IL-3 + 10 ng/ml GM-CSF). (C) Stage 3 started with the cells derived from group MNF+SFGM3 of Stage 2. Absolute numbers of total cell [C(i)], CD66b^+^ cell [C(ii)] and were calculated on day 15 in different medium formulas with G-CSF ranging from 100 to 500 ng/ml in MNF+SFG (IMDM + nutrition supplements + FBS + 100 ng/ml SCF + 100 ng/ml Flt-3L + 100 ng/ml G-CSF) medium. Results are presented as means ± SD of 6 independent experiments. One-way ANOVA followed by Dunnett’s multiple comparison tests was used for comparison among the various treatment groups.* P<0.05; ** P<0.01; *** P<0.001; **** P<0.0001; ns, No significant difference.

In Stage 1, isolated CD34^+^ cells were expanded for 6 days to expand hematopoietic stem and progenitor cells. There was no significant difference between the MNF+SFGM3T and MN+SFGM3T group in terms of hematopoietic stem cell proliferation although the total number of cells in the group MNF+SFGM3T that contained serum was higher than that of group MN+SFGM3T. We chose the MNF+SFGM3T group as the optimized one mainly based on the principle of a higher total cell number. Specifically, the MNF+SFGM3T group consisted of IMDM, nutrition supplements, 10% FBS, SCF at 100 ng/ml, Flt-3L at 100 ng/ml and TPO at 20 ng/ml, IL-3 at 25 ng/mL, GM-CSF at 15 ng/ml, and G-CSF 50 ng/ml. The proliferation folds of CD34^+^ cells and total cells were at 30 ± 2.4 and 110 ± 15, respectively. CD34^+^ cell percentage was maintained at 32% ± 4.3% throughout the culture (Fig 1A).

Stage 2 intended to evaluate various optimal cytokine combinations for differentiation toward the myeloblast lineage (Fig 1B). We selected a panel of cytokines to ensure minimal non-paracrine differentiation. For example, TPO is not necessary for neutrophil progenitor expansion [36] and its inhibitory effect on neutrophil maturation is marginal. Thus, we removed TPO in Stage 2 culture protocol. Total cell counts were higher in the group supplemented with MNF+SFGM3(GM20) (a combination of SCF, Flt-3L IL-3, G-CSF, and GM-CSF(20 ng/ml)) and MNF+SFGM3(GC75) (a combination of SCF, Flt-3L IL-3, G-CSF(75 ng/ml), and GM-CSF) cocktails than other groups supplemented with various cytokine cocktails (Fig 1B). Although MNF+SFGM3(GM20) and MNF+SFGM3(GC75) cocktails induced the highest total cell expansion the percentages of neutrophils were no difference, suggesting that a high concentration of G-CSF and GM-CSF had little effect on the induction to mature neutrophils (Fig 1B). On the basis of minimal non-paracrine differentiation and the percentage of induced neutrophils, the cocktail MNF+SFGM3 was selected for induction of differentiation and maturation of neutrophils in Stage 2. On average, after 6-day culture with MNF+SFGM3T and 3-day culture with MNF+SFGM3, the normalized yield of total cells was approximately 1.0×10^3^ cells from one CD34^+^ cell with 22.4%±4.1% of CD66b^+^ cells (n = 6, mean±SD)

**Fig 2:**
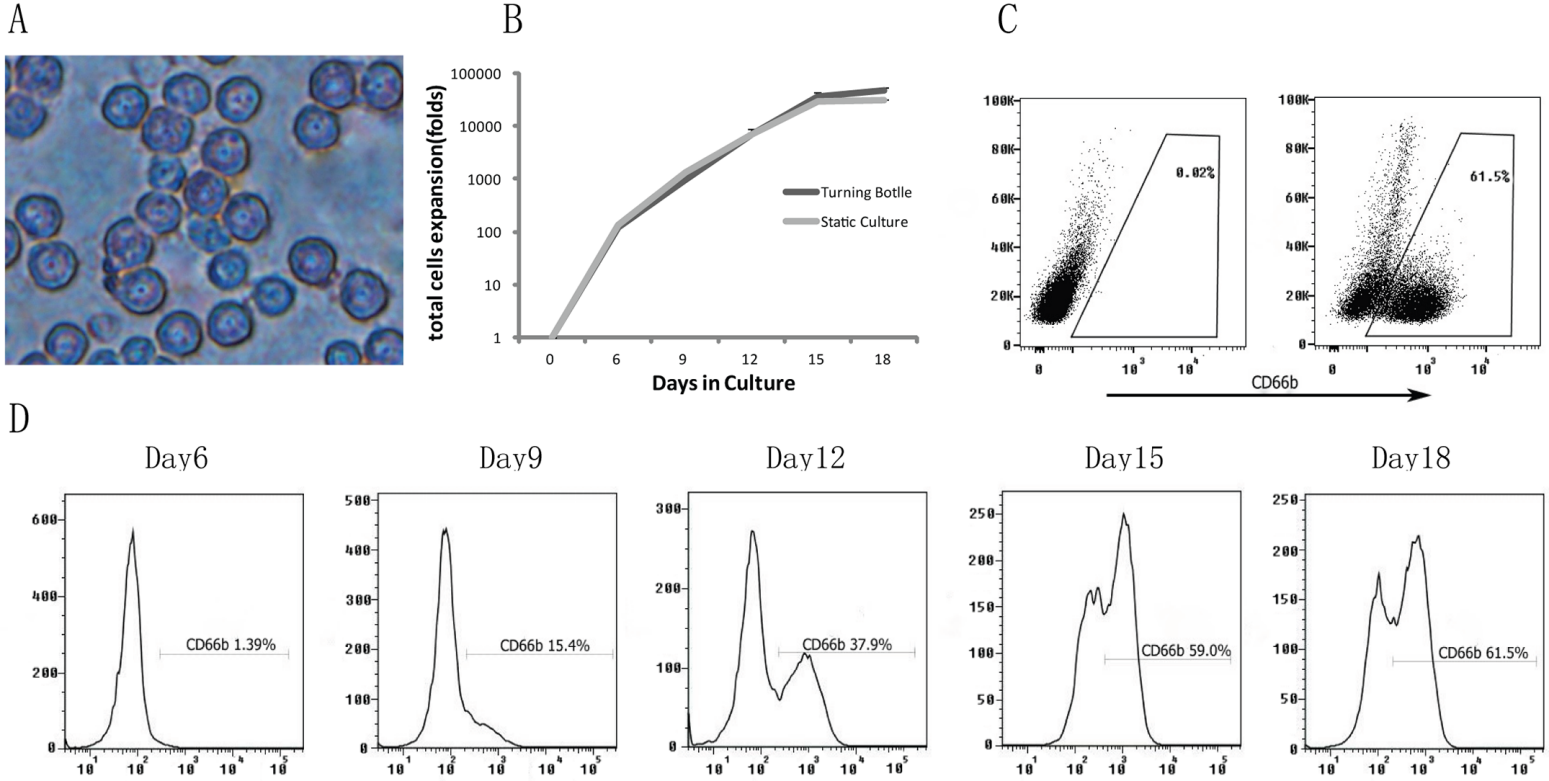
Kinetics, morphology, and characterization of differentiated neutrophils via four-stage culture system. (A) Representative images of neutrophils on day 18 after Wright-Giemisa staining (magnification ×400). (B) Isolated CD34^+^ cells were cultured in defined culture conditions. The expansion rate was calculated as fold-increase in cell counts on each day (day 0, 6, 9, 12, 15, and 18). Static culture (grey line) and turning-bottle culture (black line) expansion of human UCB CD34^+^ cells were compared. Data were collected from three independent experiments (C) Representative FACS plots for CD66b expression of human UCB-derived neutrophils on day 18. (D) Representative histograms of CD66b-positive populations during neutrophil differentiation.

In Stage 3, neutrophil cell expansion was further induced by specific cytokine combinations. In order to induce the highest neutrophil production, various combinations of G-CSF (range 100~500 ng/ml), SCF (100 ng/ml) and Flt-3L (100 ng/ml) were added to the culture medium for optimization as these cytokines were routinely used in neutrophil maturation. The results showed that G-CSF at a concentration of 100ng/ml-500ng/ml had no significant effect on neutrophil differentiation. Thus, we selected 100ng/ml G-CSF as the final concentration in the culture media (Fig 1C). We observed that G-CSF alone exhibited a low expansion potential while it synergized with SCF and Flt-3L during progenitor cell expansion (rather than only acting on differentiation and maturation of progenitor cells expanded by SCF and Flt3-L). Furthermore, morphological changes were clearly noticeable after differentiation. Mature neutrophils were multi-lobulated in shape (Fig 2A). Combined, these results demonstrate that MNF+SFG medium facilitates differentiation and maturation of human neutrophils *ex vivo* after day 9 of culture.

In Stage 4 of culture, FBS was excluded to eliminate its effect on multi-lineage differentiation. One percent of human serum albumin (HSA) was used to replace FBS as it is routinely used as an important nutrient for various cell culture processes [37, 38]. In this Stage, the total number of differentiated, mature neutrophils was amplified about 1.2 times. G-CSF appeared to extend the survival time of differentiated neutrophils likely through preventing their apoptosis [39]. Taken together, the final optimized culture medium for human neutrophil expansion and differentiation was IMDM supplemented with nutrition supplements and sequential cytokine combinations (MNF+SFGM3T for Stage 1, MNF+SFGM3 for Stage 2, MNF+SFG for Stage 3, MNA+SFG for Stage 4) which were summarized in Table 1.

### Large-scale generation of human neutrophils

With the selected cytokine combination in each stage, *ex vivo* large-scale granulocytopoiesis from human UCB CD34^+^ cells was performed in a bottle turning device culture system. As shown in the growth curve (Fig 2B), the expansion ratio of the total cells was slowly increased during the initial culture period (day 0 to day 6). From day 6 to day 12, the cells entered an exponential growth phase in which cells maintained high rates of proliferation, leading to a vigorous growth period. Approximately 8300-folds of initial cell expansion was achieved on day 12. The expansion rate slowed down from the 12th day, with a combined total cell expansion folds of 4.9×10^4^ on day18. When the culture continued, cell death was observed from day 18 (data not shown). We also used the same culture conditions (e.g., cytokine cocktails, etc) and starting cell density using the conventional stationary culture condition as a control, it is noted that more cells were obtained in the static culture conditions before the ninth day, but the number of cells in roller bottles significantly exceeded those of stationary culture conditions after day 12. In addition, the final cell number and the neutrophil percentage in the roller bottle culture system were significantly higher than those of the stationary culture at day18 (Fig 2B).

The speed of the roller bottle also had a significant impact on cell growth. A higher speed enables cells to obtain more nutrition whereas too much dissolved oxygen inhibits neutrophil maturation [40]. We found that the optimal rotational speed for expansion of human UCB differentiated neutrophils was 0.82 U.

Isolated human UCB CD34^+^ cells on day 0 express high levels of HSC markers (CD34^+^) and, as expected, low or undetectable levels of neutrophilic markers such as CD66b were detectable. The CD34^+^ ratio decreased rapidly on day 9 (3%±1%) and on day 12 (0.8%±0.3%), respectively, after 18-days of proliferation and differentiation, the percentage of CD34^+^ and CD133^+^ cells was significantly decreased to below 1%. In contrast, the percent of cells expressing CD66b rapidly increased. For example, on day 18, the neutrophil population reached 61.5% (± 5.3%) as shown in the flow cytometric histogram (Fig 2C). Percentages of CD66b^+^ cells were determined for the entire course of neutrophils differentiation and maturation (Fig 2D). In addition, differentiated and mature neutrophils were also confirmed by Giemsa staining. We observed that *ex vivo*-derived neutrophils contained intra-cellular granules in the cytoplasm and segmented nuclei (Fig 2A).

### Chemotaxis and bactericidal activities of ex vivo differentiated neutrophils (EDN) were similar to human peripheral blood isolated neutrophils (PBN)

**Fig 3:**
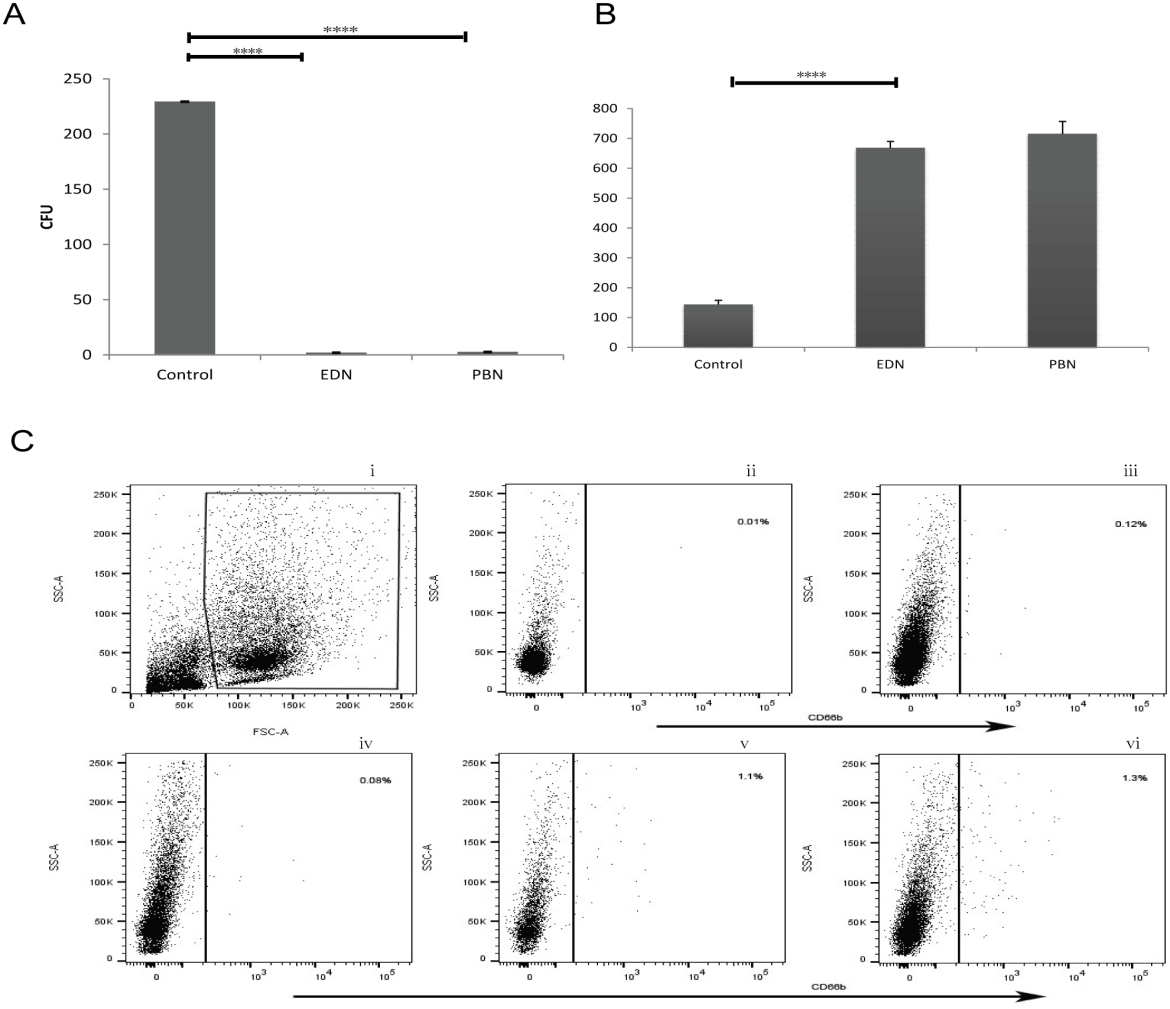
Study on bactericidal and chemotactic activities of neutrophils derived from human UCB HSC. (A) Bacterial killing assay. *E coli* was opsonized with human AB serum, and incubated with *ex-vivo* differentiated neutrophils (EDN), human peripheral blood fresh isolated neutrophils (PBN), or medium alone as a control. Bacterial colonies were significantly reduced to approximately 1% of the control after overnight incubation. There were no significant differences in the bactericidal activity between EDNs and PBNs, data were collected from three independent experiments; bars indicate SDs; One-way ANOVA followed by Dunnett’s multiple comparison tests was used for comparison among the various treatment groups. ****(P<0.0001 compared with the control). (B) Chemotactic activity of neutrophils in response to FMLP and IL-8 was determined using Transwell (3-μm pore; Corning Inc, Corning, NY 14831 USA) as described in Materials and Methods. The transwell inserts were removed after the culture plates were cultured for 2 h. Cells in the lower chamber were counted under a phase-contrast microscope. One-way ANOVA followed by Dunnett’s multiple comparison tests was used for comparison among the various treatment groups. ****(P<0.0001 compared with the control). (C) *In vivo* chemotactic activity of human UCB-derived neutrophils in the air pouch inflammatory model of NOD/SCID mice. Human UCB-derived neutrophils (v) or vehicle saline (iii) were transplanted into NOD/SCID mice intravenously. PBS (500μl) with zymosan (1 mg/ml) and IL-1β (10ng/ml) was injected into the air pouch to induce inflammation. After 16 h, cells accumulated in the pouch were collected and subjected to flow cytometric analysis for expression of neutrophil-specific marker CD66b. As a positive control, human peripheral blood isolated neutrophils (PBN) were transplanted into NOD/SCID mice as well. The air pouch inflammatory model was examined as in (vi) using both zymosan and IL-1β as inflammatory agents (Fig 3C(i). Representative dot plots were visualized according to the FSC-log and SSC-log (ii):IgG; Fig 3C (iv) shows the injection of PBS without zymosan and IL-1β but transplanted with human UCB-derived neutrophils to NOD/SCID mice intravenously followed by detecting human neutrophils in mouse peripheral blood using the same method.

In the bacterial killing assay, the negative control group was the culture with no addition of neutrophils on the coated plates, which typically yielded about 230 bacterial colonies per plate after overnight incubation. However, after co-incubation with neutrophils, regardless of EDN or PBN group, only 2–5 colonies grew on each plate, indicating that both EDNs and PBNs has an excellent bactericidal killing activity (Fig 3A). The chemotactic activity, which is one of the most critical functions of mature leukocytes including neutrophils, was further evaluated using bacterial chemotactic peptide FMLP and neutrophil specific chemokine IL-8 as chemoattractants. As shown in Fig 3B, neutrophils differentiated from human UCB displayed a chemotactic activity that was similar to neutrophils isolated from human peripheral blood. Dick et al. [21] reported that although *ex vivo*-derived neutrophils exhibit a reduced bactericidal activity *in vitro* the remaining bactericidal activity is sufficient for protection against bacterial infection, thus providing therapeutic benefits in patients with chemotherapy-induced neutropenia. These authors also noted that neutrophils with a reduced activity were likely due to specific culture conditions or improper *in vitro* assays employed [41]. Thus, it is of great interest that *ex vivo*–derived neutrophils in our distinct culture conditions exhibit the same chemotactic and bactericidal activities as those of neutrophils freshly isolated from the human peripheral blood.

### In vivo evaluation of chemotactic activity of neutrophils differentiated from human UCB

The method for evaluating chemotactic activity of neutrophils induced from human UCBs was as previously described [42]. Because of human IL-1βenhanced zymosan-induced accumulation of human CD66b-positive neutrophils, we used these two agents as chemoattractants. Sixteen hours after i.v. transplantation of human UCB-derived neutrophils and injection of proinflammatory agent(s), neutrophils accumulated in the air pouch were collected and evaluated for human CD66b-positive cells by flow cytometry. Leukocytes that accumulated in the zymosan and IL-1β induced air pouch inflammation model in normal mice were shown to be predominantly neutrophils, along with small numbers of monocytes and lymphocytes. When NOD mice were transplanted with human UCB-derived neutrophils and injected with both zymosan and IL-1β, human UCB-derived CD66b-positive neutrophils in the air pouch were 1.1% of the total accumulated cells [Fig 3C(v)], whereas human CD66b-positive cells were not significantly detected in the air pouch [31] of control NOD mice that did not receive transplantation of human UCB-derived neutrophils even when both inflammatory agents were injected [Fig 3C(iii)]. No significant numbers of CD66b-positive cells were detected in another negative control mouse group that were not injected with inflammatory agents into the air pouch but received transplanted human UCB-derived cells [Fig 3C(iv)]. When human peripheral blood neutrophils were used, the same results as those of human UCB-derived cells were obtained [Fig 3C(vi)] (i.e., human cord blood cell-derived CD66b-positive neutrophils in the air pouch of NOD mice were 1.3% of the total accumulated cells). There is no significant difference between the human UCB-derived neutrophil group and the positive control group.

### In vivo maturation of human UCB derived neutrophils in the NOD/SCID mice and long-term safety analysis

**Fig 4:**
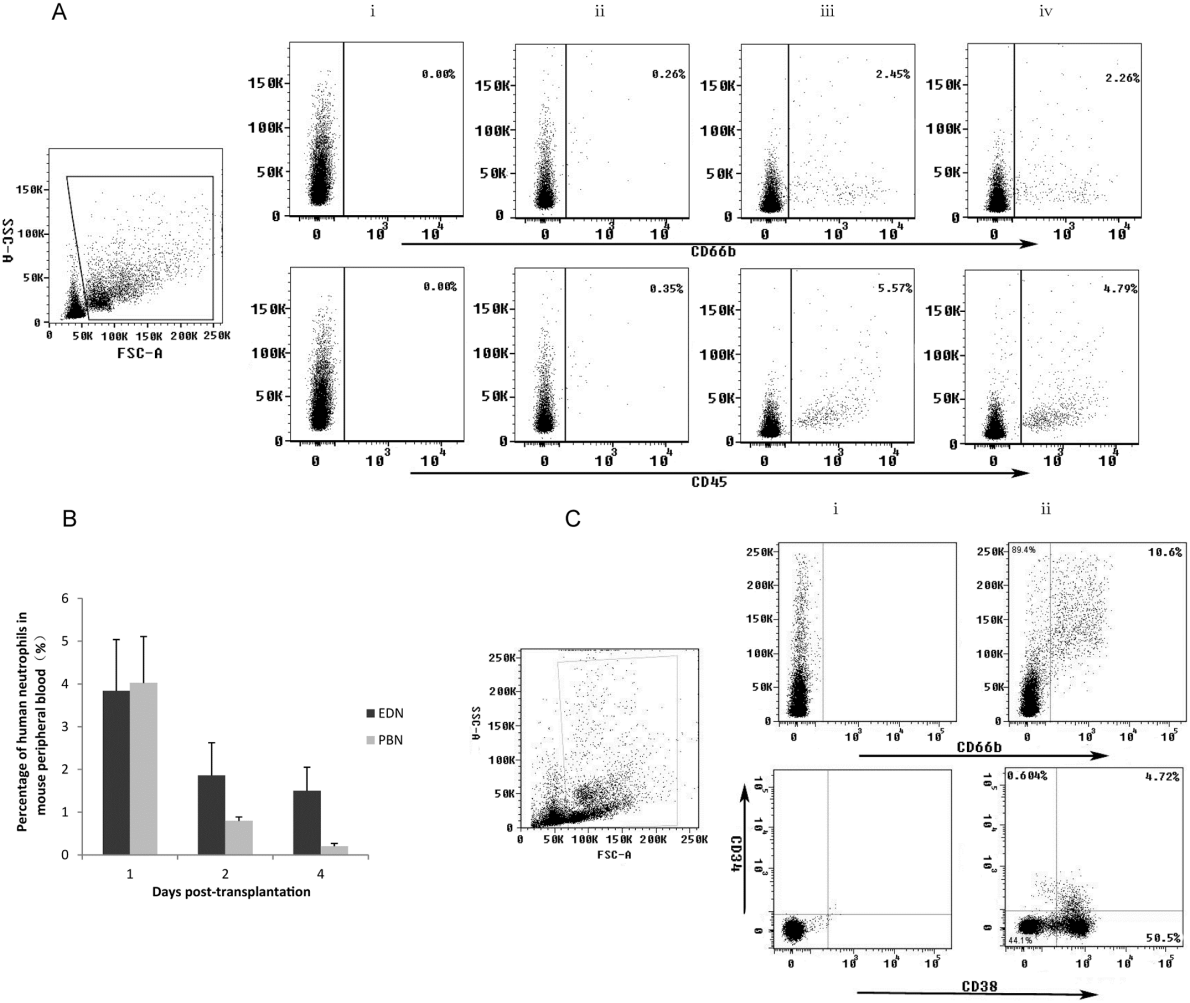
Safety and functional study of human UCB-derived neutrophils. **(A)** Human UCB-derived neutrophils (1x10^7^) and fresh human peripheral neutrophils (1x10^7^) (or saline as control), were transplanted into sublethally-irradiated NOD/SCID mice. Human neutrophils were detected in mouse peripheral blood 2 days after transplantation. Representative dot plots and flow cytometric profiles were shown. CD66b (top) and CD45 (bottom) profiles were presented for IgG (i), saline (ii), human CB-derived neutrophils (iii), and human peripheral blood neutrophils (iv) 2 days after transplantation. (B) Human UCB-derived neutrophils and peripheral blood neutrophils were detected in the mouse peripheral blood at the indicated times post-transplantation. Data are summarized as mean±SD (n = 5) at each time point. (C) *Ex-vivo*-expanded human neutrophil progenitors engrafted in the mouse bone marrow two months after transplantation. IgG (i), human UCB-derived neutrophils (CD66b^+^), promyelocytes (CD38^+^), and hematopoietic stem cells (CD34^+^) (ii). Abbreviations: FSC, forward scatter; SSC, side scatter.

The therapeutic potential to ameliorate neutropenia using human UCB-derived neutrophils remained to be determined. The first approach was to test these cells in preclinical animal models such as NOD/SCID mice. Our study showed that after *ex vivo* differentiated neutrophils (EDNs) and peripheral blood isolated neutrophils (PBNs) were injected, via tail vein, into NOD/SCID mice, human neutrophils and leucocytes were detected in the peripheral blood of transplanted mice at a percentage of 2% -5% one day after transplantation (Fig 4A). The difference between mouse groups receiving either EDNs or PBNs was mostly significant 4 days after transplantation. No human neutrophils were detected in the peripheral blood of NOD/SCID mice of the PBN group whereas 1% to 2% of human neutrophils were detected in the EDN group. The average survival period of human neutrophils was 1–2 days [43]. Thus, it was likely that human UCB-derived neutrophils continued to mature in recipient mice. CD66b^+^ cells derived from UCB survived for at least 4 more days in mouse peripheral blood (PB) after 18-day culture. On the other hand, fresh PB neutrophils lasted for only 2 days post-transplantation. These results strongly suggest that *ex vivo*-derived neutrophils/progenitors can further mature *in vivo* (Fig 4B). In fact, we continuously detected the presence of human neutrophils in mouse peripheral blood and mouse bone marrow cells contained human hematopoietic stem cells, neutrophil progenitor cells, and mature neutrophils during two months post transplantation. Percentages of human hematopoietic stem cells (CD34^+^), neutrophil progenitor cells (CD38^+^), and neutrophils (CD66b^+^) in the mouse bone marrow were 4.72%, 50.5%, and 10.6%, respectively (Fig 4C), indicating that cells (on the 9th day of culture) transplanted into the recipient mice have been successfully engrafted into the bone marrow of immunodeficient mice, leading to sustained production of neutrophil progenitors and mature cells. Transplantation of UCB-derived neutrophils elicited no adverse reactions in mice. No signs of fever, emesis, or diarrhea were observed. Moreover, 4 months after transplantation, all recipient mice survived with no apparent abnormalities.

## Discussion

It is not a routine clinical practice to collect donor neutrophils despite their potential use for supporting neutropenic patients through transfusion [44]. Major limitations for utilizing donor neutrophils for transfusion medicine including complicated processes to identify and screen donors, the necessity to mobilize HPCs with hematopoietic cytokines, and the difficulty to obtaining sufficient numbers of neutrophils even after mobilization [45]. In the current study, we have designed a four-stage culture protocol to expand UCB stem cells and differentiate them toward the neutrophilic lineage cells using the bottle turning device system with the cost-efficient culture media. The large scale *ex vivo* cell expansion system involves no gene manipulation, which is compatible with good manufacturing practice to produce neutrophils for clinical applications. Using this protocol, we can obtain 2.4x10^11^ transplant-ready neutrophils from one input human UCB CD34^+^ cells. Our yield is approximately 5-fold higher than the best one reported previously [20–22]. Theoretically speaking, neutrophils from one UCB unit (~5x10^6^ CD34^+^ cells) can be used for treating 12 or more neutropenic patients (mean weight 70 kg). Given that a large number of cells is required for transfusion, neutrophil expansion and production in standard tissue culture flasks are not feasible. Therefore, we implemented a roller-bottle culture system, which can hold up to 10 L culture medium in 16 roller bottles packed in one incubator. In fact, a sufficient number of neutrophils for one dose of clinical use can be obtained by this primary culture approach. To our knowledge, this is the first report that a bottle-turning device platform is used to scale up the production of granulocytes/neutrophils.

It has been also an issue that *ex vivo* production of a sufficient number of neutrophils for clinical applications is costly. To address this problem, we have modified the basal culture medium by the addition of nutrition supplements to IMDM so that HSCs can be better supported for expansion and differentiation. The cost of modified medium is estimated at only 1/60 (or even lower) of the StemSpan^™^ SFEM, which has been a gold-standard for a large-scale expansion or production of hematopoietic cells *ex vivo*.

We have continued to optimize culture conditions for supporting proliferation and differentiation capacity of HSCs. We have made special efforts on investigating and screening hematopoietic stem cell growth and expansion factors [46–48], as well as factors that promote differentiation toward platelets [33], red blood cells, endothelial cells [49], and neutrophils. No any gene manipulation process was involved in the culture system and we simply just used the humanized cytokines, so that the entire culture process would not affect the cell genetic stability and safety[50]. The other results from our labs show that the expression of oncogene and genes that related to the telomere is stable after efficient expansion of HSCs (33). It takes 12 days to generate mature neutrophils from myeloblasts. It has been estimated that the transition time from myeloblasts to myelocytes is about 6 days [51]. The four-stage culture protocol optimizes cytokine combinations at each stage of proliferation and/or differentiation along a particular hematopoietic lineage. We have allowed the expansion of hematopoietic stem cells in the first 6 days and differentiation of myeloid progenitors into neutrophils for the subsequent 12 days. Both SCF and Flt3-L (each at 100 ng/ml) yield a similar expansion rate when they are used alone. However, these two cytokines appear to have a synergizing effect when they used in combination [16]. G-CSF is crucial for neutrophil growth, survival, and release from the bone marrow [52, 53].

We have systematically investigated a number of cytokine combinations for *in vitro* expansion of CD34^+^ cells from human UCB. It is established that the neutrophilic lineage is established through the combined action of IL-3, GM-CSF, and G-CSF [54]. Thus, we have used these cytokines as a Stage-2 cocktail to expand and differentiate neutrophil progenitor cells. The transit time of the lineage-committed progenitors to the mature neutrophils is approximately 4 to 6 days [55] [56] [57]. Therefore, SCF, Flt-3L and optimized G-CSF concentrations have been used as a Stage-3 cocktail to stimulate the expansion of myeloblast progenitors, as well as promoting differentiation of these cells along the neutrophilic lineage [16]. With optimized culture conditions, human UCB CD34^+^ cells are efficiently driven toward the neutrophil lineage during 18-day culture. Neutrophils undergo maturation during the last 3 days of culture. We have used 1% HSA to replace FBS in addition to the presence of a cytokine combination of SCF, Flt-3L, and G-CSF. Our culture conditions ensure continued survival of mature neutrophils and in the meantime remove immunogenic components of FBS so that neutrophils obtained are compatible for clinical transplantation.

Neutrophils derived from UCB CD34^+^ cells *ex vivo* are fully functional as they exhibit chemotactic and bacterial killing effects similar to those of fresh peripheral blood neutrophils from heathy donors. *In vivo* chemotactic activity of UCB-derived neutrophils is demonstrated using an air pouch inflammatory model in NOD mice transplanted. Besides, CD66b^+^ UCB neutrophils are presented in the peripheral blood of recipient mice much longer than peripheral blood neutrophils, strongly suggesting that the *ex vivo* generated neutrophils (and progenitors) can further mature *in vivo*. To date, a few research groups have investigated *ex vivo* expansion of neutrophil progenitors from HSCs [58–60], and in two such studies, expanded cells have been used clinically for providing autologous HSCs. In the current study, we have injected cryopreserved neutrophil progenitor cells (day 9 culture) into neutropenia NOD/SCID mice via the tail vein. Human mature neutrophils in the mouse peripheral blood are capable of sustaining for a period of time as we have detected the presence of human neutrophil progenitor cells and mature cells in the mouse bone marrow two months after injection, which provides new insights into clinical applications. *Ex vivo* expanded neutrophil progenitors can provide not only a temporary relief for neutropenic patients but also a long-term support for myeloid progenitor deficiency in the bone marrow. Furthermore, the neutrophil progenitor cells can be cryopreserved, thus making their storage and transportation easy. Of note, mice transplanted with human UCB-derived neutrophils survive well for a long time and no apparent abnormalities including tumor development are observed.

Taken together, we have established a pilot-scale culture system to produce functional human neutrophils *ex vivo*. Considering that one neutrophil transfusion unit (100 ml) contains 2×10^10^ cells, the CD34^+^ cells from one UCB unit (80 ml) will generate 2.4×10^11^ neutrophils, which are equivalent to 12 units/doses of neutrophils for clinical transfusion. Given excellent features associated with the neutrophils derived from UCB, we believe that these cells can be used as an alternative source to conventional neutrophil transfusion in the clinic.

## Supporting information

S1 Fig Kinetics of CD34^+^ hematopoietic stem/progenitor cell on stages 1 and 2 of culture. Isolated CD34^+^ cells were cultured with selected culture conditions, representative dot plots of CD34 cell-surface markers on uncultured cells(day0) and expanded cells (day6 and day 9).
(DOCX)
