# Supplementary material for: Large-scale ex vivo generation of human neutrophils from cord blood CD34+ cells
Source: PLoS One. 2017 Jul 11;12(7):e0180832. doi: 10.1371/journal.pone.0180832 (PMC5507460; doi:10.1371/journal.pone.0180832)
Supplement: S1 Fig — Isolated CD34+ cells were cultured with selected culture conditions, representative dot plots of CD34 cell-surface markers on uncultured cells(day0) and expanded cells (day6 and day 9). (DOCX) [file pone.0180832.s001.docx]

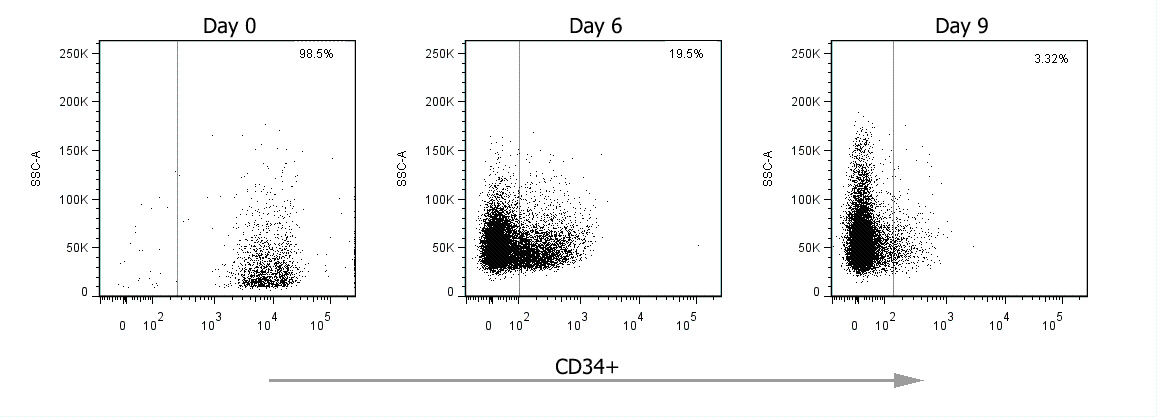


**Fig. S1. Kinetics of CD34^+^ hematopoietic stem/progenitor cell on stages 1 and 2 of culture.** Isolated CD34^+^ cells were cultured with selected culture conditions, representative dot plots of CD34 cell-surface markers on uncultured cells(day0) and expanded cells (day6 and day 9).
